# Effect of DNA methylation inhibitor on RASSF1A genes expression in non-small cell lung cancer cell line A549 and A549DDP

**DOI:** 10.1186/1475-2867-13-91

**Published:** 2013-09-08

**Authors:** Duan Mengxi, Wang Qian, Wang Nan, Xiao Xiaoguang, Li Shijun

**Affiliations:** 1 Department of Clinical Laboratory, The First Affiliated Hospital of Dalian Medical University, Dalian 116011, China

**Keywords:** RASSF1A, DNA methylation, 5-Aza-2′-deoxycytidine, Non-small cell lung cancer, Resistance

## Abstract

**Background:**

Ras association domain family 1A gene (RASSFlA) is a candidate suppressor gene, Lack of RASSF1A expression was found in lung cancer. High DNA methylation at the promoter region is the main reason for inactivating RASSF1A transcription.

**Methods:**

In this study, we examined RASSF1A’s methylation status and its mRNA expression level between non-small cell lung cancer cell line A549 and anti-Cisplatin cell strain A549DDP, Furthermore, methylation of A549DDP was reversed by treatment of 5-Aza-2′ - deoxycytidine (5-Aza-cdR),a DNA methyltransferase inhibitor.

**Results:**

We found that RASSF1A’s methylation status and its mRNA expression were obvious differences between A549 and A549DDP. 5-Aza-CdR treatment remarkablly reduced cell vability of A549DDP. Moreover, 5-Aza-CdR treatment induced A549DDP cell apoptosis in a dose dependent manner with declining cell percentage in S and G2/M stage, and increasing proportion in G0/G1 stage. Cell motility was blocked in G0/G1 stage. All of A549DDP cells showed unmethylated expression, its high methylation status was reversed in a dose-dependent manner within a certain range.

**Conclusions:**

The abnormal gene methylation status of RASSF1A is a molecular biomarker in lung cancer diagnosis, treatment and prognosis.

## Introduction

Lung cancer is one of the most common malignant tumors with highest incidence and morbidity [1,2]. Late detection is a major contributor to these high mortality rates [3]. Most patients are already at the late stage of lung cancer when they are diagnosed. Cisplatin [4] is the first-line chemotherapy drug, used in the late stage of lung cancer. However, cisplatin and other drugs are not efficient chemotherapy in non-small cell lung cancer (NSCLC). It is the most urgent to conquer the drug-resistance of NSCLC in cancer therapy.

Recent studies have shown that the inactivation of tumor-suppressor genes plays an important role in the pathogenesis and chemotherapy sensitiveness of lung cancer [5]. Ras association domain family 1A gene (RASSFlA) is a candidate suppressor gene [6], which is localized at 3p21.3, coding a group of RAS protein. Homozygote and heterozygote loss of RASSF1A was found in more than 90% of small cell lung cancer and in 50-80% of non-small cell lung cancer [7,8]. High DNA methylation at the promoter region is the main reason [9], especially in CpG islands. Low methylation of DNA can make chromosome instability [10], such as chromosome breakage, translocation and lost, and proto-oncogene activation; High methylation of DNA promoter region CpG islands can make the tumor suppressor gene inactivation [11,12]. Methylation status is a biomarker, but it can be reversed by methyltransferase inhibitor, such as 5 - Aza – CdR [13]. In this study, we studied the abnormal gene methylation status of RASSF1A in NSCLC cell line A549 and anti-Cisplatin cell strain A549DDP and suggested that methyaltion of RASSF1A is a molecular biomarker in cancer diagnosis, treatment and prognosis.

## Results

### RASSF1A methylation in lung cancer cells

**Figure 1:**
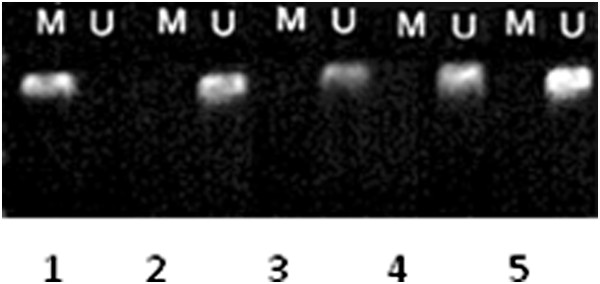
**MSP detected RASSF1A’s methylation status before and after adding to 5 -Aza –CdR in different concentration.** Lane M: methylated stripe (119 bp); Lane U: unmethylated stripe (125 bp); Lane 1: A549DDP cell; Lane 2: A549 cell; Lane 3: A549DDP 5 μmol/L group; Lane 4: A549DDP 10 μmol/L group; Lane 5: A549DDP 20 μmol/L group.

To investigate the different status of RASSF1A DNA methylation in lung cancer cells, we examined DNA methylation of RASSF1A in the Cisplatin-resistant cell strain A549DDP and A549 lung cancer cells by MSP. We found that RASSF1A gene was mostly methylated in Cisplatin-resistant cell strain A549DDP; but, mostly unmethylated in A549 cell line (Figure 1).

### RASSF1A mRNA expressionin in lung cancer cells

**Figure 2:**
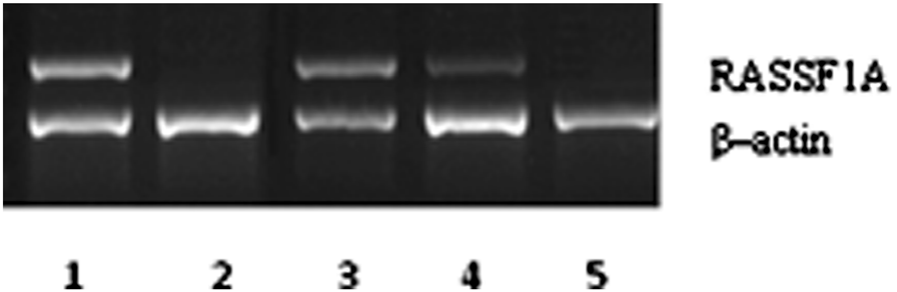
**RASSF1A gene’s expression detected by RT-PCR.** Up Lane: RASSF1A (331 bp); Down Lane: β–actin (271 bp), Lane 1: A549 cell; Lane 2: A549DDP cell; Lane 3: A549DDP 5 μmol/L group; Lane 4: A549DDP 10 μmol/L group; Lane 5: A549DDP 20 μmol/L group.

To investigate the different of RASSF1A gene’s expression between A549DDP and A549 lung cancer cells,we used RT-PCR to detect it. We found that RASSF1A gene’s expression in A549 cell line was clearly higher than A549DDP. In these two kinds of cells, the RASSF1A methylation status was tended to vary inversely with its mRNA’s transcriptional level (Figure 2). We hypothesized that DNA methylation can cause gene expression to cut down.

### 5 -Aza -CdR can effect RASSF1A gene methylation status and gene expression

We found that RASSF1A’s methylation status and its mRNA’s expression were obvious differences between A549 and A549DDP. In A549DDP group, RASSF1A gene was highly methylation expression and little mRNA expression. Wether such status can be reversed by methyltransferase inhibitor, we added different concentration of 5 - Aza - cdR to A549DDP cells. After training 48 hrs, we found that RASSF1A genes showed unmethylated states and the mRNA expression increased in all experimental groups. Used optical density ratio of β-actin gene, when the 5-Aza-CdR concentation was 0 μmol/L, 5 μmol/L, 10 μmol/L, 20 μmol/L, the relative expression level were 0.06 ± 0.02, 0.22 ± 0.11, 0.36 ± 0.16, 0.54 ± 0.15. The RASSF1A mRNA expression was raised in each group, the difference had statistics significance (P < 0.05) (Figures 1 and 2).

### Detecting A549DDP cell inhibitory situation by MTT assay

**Figure 3:**
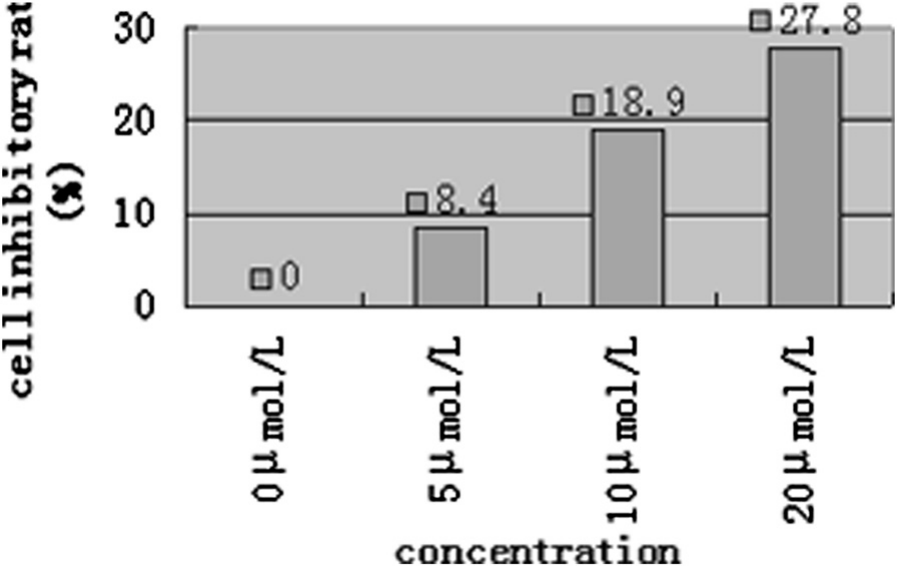
MTT assay detected A549DDP cell inhibitory situation after 48 hrs intervention of 5-Aza-CdR in different concentrations.

To investigate the different cell inhibitory status between lung cancer cells, we examined them by MTT assay. We found that A549DDP cells showed a remarkable inhibition action after 48 hrs treatment of 5-Aza-CdR. The inhibition rate increased with the increase of drug dosage in a dose dependent manner. The inhibition rates were 8.4%, 18.9%, 27.8%, when drug dosage were 5 μmol/L, 10 μmol/L, 20 μmol/L,which was statistically significant by comparison with control group (P < 0.05) (Figure 3). We hypothesized that 5-Aza-CdR treatment remarkablly rduced cell vability of A549DDP.

### Cell apoptosis and cell cycle detected by flow cytometry

**Figure 4:**
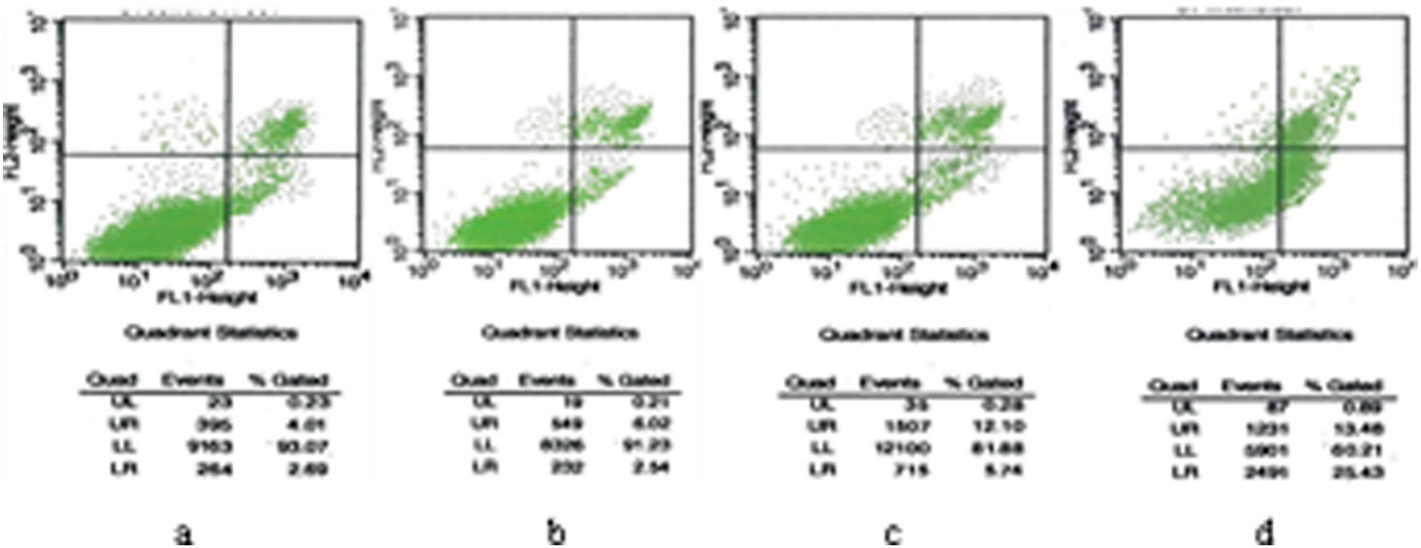
**Cell apoptosis detected by flow cytometry with FITC and PI staining.** LL shows living cells (FITC-/PI-); UR shows necrotic cells (FITC+/PI+); LR shows apoptotic cells (FITC +/PI-); **a:** control group (0 μmol/L 5-Aza-CdR), **b:** 5 μmol/L 5-Aza-CdR, **c:** 10 μmol/L 5-Aza-CdR, **d:** 20 μmol/L 5-Aza-CdR.

To investigate the different cell apoptosis and cell cycle between lung cancer cells, we examined them by flow cytometry. We found that after 48 hrs intervention of 5-Aza-CdR, A549DDP cells apoptosis increasing, and go with the increase of drug dosage in a dose dependent manner. When concentration were 0 μmol/L, 5 μmol/L, 10 μmol/L, 20 μmol/L, the apoptosis rate were 6.70%, 8.56%, 17.84%, 38.91%. The results of 10 μmol/L and 20 μmol/L goupe were statistically significant by comparison with control group (P < 0.05), but 5 μmol/L group had no statistically signification (P > 0.05). (Figure 4). At the same time, cell percentage in S and G2/M declined, and proportion in G0/G1 stage increased. Cell motility was blocked in G0/G1 stage (Table 1).

**Table 1:** Cell cycle detected by flow cytometry

**Group**	**G0/G1**	**S**	**G2/M**
Control	60.94 ± 1.73	34.12 ± 1.52	4.94 ± 0.27
5 μmol/L	62.79 ± 2.21	31.82 ± 1.77	5.39 ± 0.36
10 μmol/L	69.35 ± 1.46	26.41 ± 1.67	4.24 ± 0.83
20 μmol/L	79.98 ± 2.08	18.07 ± 1.03	1.95 ± 0.53

## Discussion

RASSF1A is a tumor suppressor gene in widely research, that participation in a variety of biological events, such as cell growth, differentiation and apoptosis, plays an important inbibitional effect in tumor growth process, RASSF1A has also been associated with stabilization of microtubules and has been shown to influence cell motility and genomic stability [14-16]. RASSF1A promoter methylation states in small cell lung cancer, non small cell lung cancer and breast cancer cell lines were 100%, 63% and 64% respectively,while the nomal lung tissue is none, and higher degree in poorly differentiated tumors, but lower in well differentiated tumors [17]. Dammann [18] studied eight pancreatic cancer cell lines found that RASSF1A gene promoter regions methylated in almost seven lines, five lines were completely methylated, two were partly methylated. So DNA methylation can result in tumor occurrence, development and drug-resistant, it has become a new research direction.

In the study, RASSF1A mainly unmethylation status in A549 cells, but high methylation status in A549DDP; And its mRNA expression of A549 was obviously higher than that of A549DDP. The relationships between them had inversely proportional. It was showed that high promoter methylation status of RASSF1A might be an important foundation to reduce gene transcription activity, moreover involved in drug resistance in non-small cell lung cancer. 5 - Aza - CdR can make RASSF1A gene to demethylate and gene expression again or enhance. Further showed that it also significantly inhibited A549DDP cell proliferation and increased cell apoptosis rate, cell cycle arrest in G0/G1 phase.

As DNA methyltransferase inhibitor, 5 - Aza - CdR [19] mainly by phosphorylated action, and do not affect DNA synthesis, restore normal function, which is very important to cell differentiation and proliferation. Mostly important, 5 - Aza - CdR is insensitive to non-proliferative cells. The Federal Drug Administration has approved DNA methyltransferase inhibitors for the treatment of myelodysplastic syndrome [20]. Drugs of this class have also been used successfully in other hematological malignancies [21]. More recently, pre-clinical and clinical data have demonstrated that these inhibitors can be used to reduce the malignant potential of solid tumors as well [22]. But 5 - Aza - CdR is not stable and has toxicity in aqueous solution. The demethylation is lack of specific target. It is worth to further discussing that its clinical efficacy, tolerability in vivo and the best compatibility dose, if it can be used in lung cancer treatment.

## Conclusions

Taken together, we demonstrate that RASSF1A’s methylation status was reversed by treatment of 5-Aza-2′- deoxycytidine. Moreover, cell apoptosis and growth were changed. Cell motility was blocked in G0/G1 stage. RASSF1A’s methylation status may be a new biology marker and gene therapy target in lung cancer.

## Materials and methods

### Cell culture

Human non-small lung cancer cell line A549 obtained from the First Affiliated Hospital of Dalian Medical University (Liaoning, China). Human anti-Cisplatin non-small lung cancer cell strain A549DDP Purchased in Runcheng biotechnology company (Nanjing, China). A549 and A549DDP cells were cultured in RPMI-1640 medium (Invitrogen, USA) containing 10% fetal bovine serum (Gibcobrl, USA), at 37°C in a humidified incubator containing 5% CO2. Added cisplatin (Jiangsu Haosen, China) to A549DDP medium and the final concentration was 2 μg/ml; The cells were passaged once or twice every week. Cells at algorism growth phase were used in following experiments.

### Methylation-specific PCR(MSP)

Both cells were collected and washed with phosphate buffered saline (PBS). Adjusted cell number to 2 × 10^3^. DNA was extracted from the cells using EZ DNA Methylation-Direct Kit™ (ZYMO, USA) according to the manufacturer’s protocol. Reaction system content 10 μl Tag PCR Master Mix, 1 μl Primer1, 1 μl Primer2, add ddH_2_O to 20 μl. Upstream and downstream primers for RASSF1A-M (methylated stripe, 119 bp) were 5′-GTGTTAACGCGTTGCGTATC-3′ and 5′-AACCCCGCGAACTAAAAACGA-3′, respectively. Upstream and downstream primers for RASSF1A-U (unmethylated stripe, 125 bp) were 5′-TTTGGTTGGAGTGTGTTAATGTG-3′ and 5′-CAAACCCCACAAACTAAAAACAA-3′. The conditions were 94°C for 5 min, followed by 35 cycles of 94°C for 30s, 55°C (RASSF1A-M) or 60°C (RASSF1A-U)for 30 s, and 72°C for 30s and a final extension at 72°C for 10 min. 10 μl PCR products were separated on the 2% agarose gel and viewed by ethidium bromide (EB) staining. By using tanon image analysis software, scaned optical density of RASSF1A and β-actin gene in electrophoresis image, indicating with both absorbance ratios.

### RT-PCR

Both cells were collected and washed by phosphate buffered saline (PBS). Total RNA was extracted from the cells using RNAiso reagent (Takara, Dalian, China) 1 μl total RNA were amplified by using PrimeScript™ One Step RT-PCR Kit Ver.2 (Takara, Dalian, China), reaction system was 50 μl, according to the manufacturer’s protocol. Upstream and downstream primers for RASSF1A (331 bp) were 5′–ACTTGCGGAAGCTGTTGGA-3′ and 5′-CGCTGCAGGATACG TAGGAA-3′, respectively. Upstream and downstream primers for β-actin (271 bp) were 5′-CTACAATGAGCTGCGTGTGGC-3′ and5′-CAGGTCCAGACGCAGGATGGC-3′, respectively. The conditions were 50°C for 30 min, 94°C for 2 min, followed by 30 cycles of 94°C for 30 s, 60°C for 30 s, and 72°C for 1 min. 10 μl PCR products were separated on the 2% agarose gel and viewed by ethidium bromide (EB) staining. These data were acquired with Tianneng software too.

### A549DDP cells treatment by 5 - Aza – cdR in different concentration

A549DDP cells in logarithmic phase with cell number was (1.5-2) × 10^4^/ml were incubated in a 96-well plate, each hole for 200 μl, at 37°C in a humidified incubator containing 5% CO2 to continue training. Removed the nutrient solution after 24 hrs, and then, added to different concentration of 5 - Aza - cdR (5 μ mol/l, 10 μ mol/l, and 20 μ mol/l) respectively, final volume was 200 μl/well each hole. The control group was the holes that without adding drug. Each group had four Repeat holes, further training at 37°C for 48 hrs.

### 5-Aza-CdR treatment effecting RASSF1A’s methylation status and its mRNA’s expression

Extracting DNA of above drug treated cells for MSP, total RNA for RT-PCR, to detect the change of RASSF1A’s methylation status and mRNA’s expression level after adding 5 - Aza - CdR. The conditions and methods follow the former.

### MTT assay

The above A549DDP cells in each group were added to 5 mg/ml MTT (20 μl/well) and further incubated at 37°C for 4 hrs, then added with DMSO (150 μl/well) and incubated for 10 min. A592 was determined by a 96-well microtitre plate reader. According to the following formula, all experiments were performed in triplicate.

$$\begin{array}{l} \text{Cell}\;\text{inhibitory}\;\text{rate}\;\left(\%\right)\\ \;=\left(1–\mathrm{ODof}\;\text{test}\;\text{group}/\mathrm{OD}\;\mathrm{of}\;\text{control}\;\text{group}\right)\\ \;\times 100\%. \end{array}$$

### Flow cytometry

A549DDP cells above were collected, washed three times with PBS and added to 1 ml ethanol (70%), fixed 2 hrs at 4°C. Washed with PBS, and then, removed supernatant after centrifugating. For detecting cell cycle, added with 0.5 ml propidium iodide (PI), then incubated at 37°C in dark for 30 min; For detecting cell apoptosis rate, added with 500 μl Annexin binding buffer, 5 μl Annexin V-FITC, and 5 μl propidium iodide (PI), then incubated at room temperature in dark for 15 min. The cell apoptosis and cell cycle were determined by flow cytometry (BD, USA) and analyzed with Cell Quest software.

### Statistical analysis

Statistical analysis was performed using SPSS 13.0 software (SPSS Incorporation, Chicago, USA). Data are expressed as mean ± standard deviation (SD) and analyzed by ANOVA. P < 0.05 was considered statistics significance.

## Abbreviations

RASSFlA: Ras association domain family 1A gene; RT-PCR: Reverse transcription-polymerase chain reaction; 5-Aza-cdR: 5-Aza-2′ - deoxycytidine; NSCLC: Non -small cell lung cancer; PBS: Phosphate buffered saline; MSP: Methylation-specific PCR; EB: Ethidium bromide.

## Competing interests

The authors declare no conflict of interests.

## Authors’ contributions

LS is corresponding author and organized the study, DM and WQ analyzed data, performed experiments and drafted the manuscript. WN and XX coordinated the study, participated in its design. All authors read and approved the final manuscript.
